# The COVID‐19 BreastScreen Department – beyond the pandemic

**DOI:** 10.1002/jmrs.430

**Published:** 2020-10-07

**Authors:** Kelly Maree Spuur

**Affiliations:** ^1^ Faculty of Science School of Dentistry & Health Sciences Charles Sturt University Wagga Wagga New South Wales Australia

**Keywords:** Breast imaging, breast screening, COVID‐19, mammography, pandemic, positioning, radiographer

## Abstract

The first wave of the COVID‐19 pandemic in Australia forced a temporary closure of BreastScreen Australia services. Now reopened, the BreastScreen experience has been redefined for both staff and clients and the journey to the ‘new BreastScreen normal’ is continually evolving in response to the ongoing threat of COVID‐19 and government directives on health policy. Many changes mirror those undertaken in the wider community and emphasise wellness to attend, hygiene and social distancing. Importantly, radiographers have been identified as having a high‐risk role and have had to modify positioning techniques and cleaning regimes accordingly. Beyond the pandemic, the ‘new normal’ needs to be one which enables well women to continue screening with a visible sense of reassurance that all that can be done is being done to ensure the safe and continued early detection of breast cancer.

## Graphical abstract

BreastScreen Australia has had to respond directly to the COVID‐19 threat to protect its staff, clients, their families and the wider community, making changes to the way it operates including a never before seen shut down services. COVID‐19 has redefined a ‘new BreastScreen normal’ with key changes essentially mirroring those undertaken in the wider community in an effort to limit the transmission of COVID‐19 and more specific changes impacting imaging. As policy and procedures continually evolve in response to the ongoing threat, the ‘new normal’ needs to be one which enables well women to continue screening with a visible sense of reassurance that all that can be done is being done to ensure the safe and continued early detection of breast cancer.

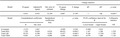

## Introduction

BreastScreen Australia (BSA) imaged more than 1.8 million women in 2016–2017.^1^, ^2^ Although breast screening is an elective examination in an asymptomatic population, Australian women have continued to actively engage the preventative health behaviour and participate in the free government breast screening programme despite the ongoing threat of COVID‐19. COVID‐19 has however extensively impacted BSA and like all providers of medical imaging worldwide, BSA has had to respond directly to the COVID‐19 threat to protect its staff, clients, their families and the wider community by making changes to the way it operates, including a never before seen shut down of services.^3^

In the initial stages of the pandemic, COVID‐19 is known to have impacted attendance for screening with the fear of infection seeing some women cancel their appointments or postponing them in an effort to ‘do the right thing’ and adhere to the ‘stay‐at‐home’ message put forward by the Australian government.^4^ At the same time, professional bodies such as the Royal Australian and New Zealand College of Radiologists (RANZCR), the Breast Surgeons of Australia and New Zealand (BreastSurgANZ Council) and the Australian Society of Medical Imaging and Radiation Therapy (ASMIRT) were also reacting to the threat, advocating for the cessation of routine screening.^5^, ^6^

Despite concern that without screening, pre‐clinical breast cancers would remain undiagnosed, the RANZCR argued that COVID‐19 is a significant risk to the older screening demographic who face greater adverse outcomes from COVID‐19 than the general population. The RANZCR put forward that even a delay of 6–12 months would not see a significant progression of an early undetected breast cancer.^6^ The view was that to continue screening was an unnecessary risk to all involved and called for an immediate cessation of screening.^6^

These views were ultimately agreed to by BSA, determining that the ongoing risk involved with continuing screening outweighed the benefits and routine screening was suspended.^7^ However, ensuring continuity of care, recently screened women were still notified of their results and women requiring an appointment at an assessment clinic were able to attend for additional testing.^7^ In due course, it was announced that all sites and services were closed.

As the measures imposed by the Australian government flattened the curve associated with the first COVID‐19 wave, BSA reopened, all be it to a ‘new normal’. The reopening process has ultimately been a direct reflection of the COVID‐19 threat in each community with advice from BSA being that resumption would be varied by location, with no specific date for when all services would resume.^7^ Some states were advising that not all screening clinics were open and those that were may have reduced bookings to best mange social distancing and cleaning.^8^ Others that screening services would recommence slowly through a planned staged approach.^9^ With a second wave of infections now affecting some States, it has become evident that the ‘new BreastScreen normal’ will involve ongoing disruption to services and continued changes to practice, the extent being more severe in some jurisdictions than others.

## Changes to bookings and clinics

All non‐clinical changes in the BreastScreen setting essentially mirror those undertaken in the wider community. In an effort to limit the transmission of COVID‐19, women are encouraged to reschedule if they have recently travelled overseas or more currently to hotspot locations; have COVID‐19 like symptoms; have been diagnosed with COVID‐19 or have been in contact with a confirmed COVID‐19 case^8^, ^9^, ^10^, ^11^, ^12^. Some services are also prioritising bookings for due or overdue women in the target age group (50–74 years).^12^

Women are also informed that key safety measures are in place at their appointment site. However, reflective of each State and Territory’s COVID‐19 status, not all Services provide the same level of detail with the NT BreastScreen website having only a generic link to Corona virus news, updates and alerts,^13^ BreastScreen Queensland’s site stating that’… Queensland services are implementing a range of strategies to minimise and address risks from COVID‐19 for women who attend for breast screening and our staff’.^2^ and BreastScreen WA that they ‘...will continue to ensure the highest levels of hygiene and care’.^14^ The level of information available appears directly proportional to the risk of COVID‐19. Other states such as NSW and Victoria who are at the forefront of a second COVID‐19 wave have provided more extensive and detailed information.^8^, ^15^

The burden of managing the important safety measures of risk profiling, temperature checks and social distancing at appointments has largely become the responsibility of clinic reception staff. Where women are able to adhere to requests to only attend their appointment 5 minutes before scheduled^8^, ^15^ and alone, waiting rooms have almost become ‘no waiting rooms’ as a one in one out ‘don't come early, don't come late’^10^ schedule is aimed for to minimise the number of people at the clinic, their time in the clinic and to maximise social distancing. This is not always possible where women are brought by taxi, community transport (they may arrive early or late) or are accompanied by careers or require translators.^8^

## Screening

Whilst working from home has enabled much of the Australian population to manage social distancing, the technical requirements of mammographic imaging necessitate that radiographers attend the workplace and work one on one with clients.

The World Health Organization (WHO) reports that COVID‐19 is primarily transmitted between individuals through respiratory droplets from a person in close contact (within 1m) and by indirect contact with surfaces.^16^ Because of these transmission routes, radiographers are at high risk as the procedure requires close (face‐to‐face) contact with the client.^15^ ASMIRT advocated that any resumption of screening give due consideration to the close client radiographer proximity^17^ (~23–42 cm) and the large client volume (~27–35 women per day) associated with routine screening.

## Changes to Imaging

Infection control management during COVID‐19 has demanded changes to the way imaging is undertaken. BreastScreen NSW informs women that minimal face‐to‐face contact will be managed by radiographers under NSW Standard Health Precautions.^15^ Although there has been no change to the routine imaging series required (craniocaudal (CC) and mediolateral oblique (MLO) views), the way in which the procedure itself is undertaken has changed for some radiographers.

There is no mandated or evidence‐based approach to radiographer positioning in mammography. The literature and educational texts vary and as such the approach to positioning is wide‐ranging between individual radiographers, sites and services. Therefore, the changes to positioning necessitated by COVID‐19 have not presented the same challenge to all radiographers.

Positioning for the CC view poses the greatest risk. In this view, radiographers may position from the woman’s medial or lateral side, with the woman’s head turned away from the breast being imaged. Where a radiographer positions from the medial side, the proximity of their face to the client is close, ~23cm. For a radiographer working from the lateral side, this distance is significantly increased to over 40cm. Differences between the height of the client and radiographer will also impact this distance. Some radiographers also employ a breathing technique requiring the woman to exhale with the aim relaxing the upper body allowing more posterior breast tissue onto the receptor. This action is also said to increase the potential for the pectoralis major muscle to be included in the image; however, there is no evidence base to support this. Although time spent positioning is limited, the radiographer who positions from the medial side and or uses the exhalation technique is at an increased risk of airborne infection.

To overcome these issues in NSW, advice was given to position exclusively from the lateral side of the breast being imaged and to cease the exhale technique.^18^ Similar advice was put forward by ASMIRT.^17^ For those radiographers who have had to modify their technique, there is to be an expected impact on image quality whilst adapting to the recommendations. How significant the impact maybe is yet to be analysed, but it should not be expected to impact cancer detection.

In the MLO view, the client to radiographer face‐to‐face distance varies depending on radiographer client height and whether the radiographer positions standing or seated and the approach to positioning (anteriorly or posterior to anterior). Distance can be further managed to the radiographers’ advantage and with no impact to the resultant image by asking the client to look upwards.^18^ Compliance with this change is easily achieved. It should be noted though that some women in the demographic may have degenerative change that may limit neck movement. More recent requirements with the second wave in some jurisdictions for both clients (if possible) and staff to wear masks where physical distancing (providing care within 1.5 m of clients) cannot be maintained, such as mammographic imaging, will also help minimise the spread of the virus.^19^

## Changes to equipment cleaning

The provision of quality healthcare services, including those of BSA is founded in best practice infection control for clinical practice through national infection control standards and legislation governing infection control and occupational health.^20^ BSA National Accreditation Standards (NAS) state that ‘equipment should adhere to the manufacturer's recommendations and/or suitable infection control advice and include the cleaning of breast support and compression paddle between each examination to meet relevant state, territory and national standards’.^20^

COVID‐19 cleaning has expanded this to include areas of the mammography unit not in direct contact with the client. Reductions in client numbers to accommodate the additional time required for cleaning have been necessary.^8^ Radiographers must be mindful that their cleaning regimes do not damage to the electronic components or the plastics, aluminium or carbon fibre of the unit, and in particular, liquids must not be allowed to enter the internal parts of the equipment.^21^

Anecdotally, women appear satisfied that adequate steps have been taken to ensure their safety within the limitations of the same. Moving forward, the challenge of balancing the benefits of breast screening and the expectations of the screening population with the potential for clients and staff to be exposed to COVID‐19 are significant. Some believe that the risk outweighs the benefits and that it is safer to reduce the risk of spreading COVID‐19 and avoid screening all together.^22^ The Australian Government Department of Health continues to monitor the impact of the COVID‐19 pandemic on its health services including BSA.^3^ For now, women remain engaged with preventive health behaviour shaped by perceived risk, well communicated messages of safety and sound infection control procedures.

As a second wave of COVID‐19 infections emerges, the threat of ongoing disruption (suspension of routine screening or closures) remains real. There is a great concern that due to the BSA closedown, some cancers may have already been missed, as women either do not reschedule or further delay their appointment. A decrease in the number of breast cancers detected has already been seen in data from the Cancer Council; simply some women are not getting tested. In Victoria during the months of April and May 2020, a 37% reduction in the number of breast cancers diagnosed in either the BreastScreen or diagnostic setting has been reported.^23^ There is significant concern that the trend will continue particularly in areas where there is reimposition of lockdown.

## Conclusion

In a time of uncertainty, some routine is important and this is especially so with respect to breast cancer detection. Without successful eradication of or a vaccine for COVID‐19 the ‘new BreastScreen normal’ will be a reflection of COVID‐19 risk minimisation. Whilst most radiology departments continue to experience fluctuation and downturn in client throughput, BSA is expecting to return to full pre‐COVID‐19 capacity in the near future. However, by necessity, BSA operations must remain both flexible and responsive to the threat. The ‘new normal’ for screening services therefore needs to be one which enables well women and staff to continue screening with a visible sense of reassurance that all that can be done is being done to ensure the safe and continued early detection of breast cancer.
